# Special Interview with Mr. Conrad W. Thies

**Published:** 1911-05-20

**Authors:** 


### STATE INSURANCE.

### SPECIAL INTERVIEW WITH MR. CONRAD W. THIES.

So much anxiety has been felt as to the ultimate effects on hospitals of Mr. Lloyd George's proposals embodied in his Insurance Bill, that the opinion of those on whom rests the responsibility of raising the voluntary hospitals' income every year becomes of the first importance. We know of no one to whom practical experience has given a greater right to speak than to Mr. Conrad Thies, for so many years secretary to the Royal Free Hospital, and his views, and concluding suggestion, will be read with the greatest interest. It is only an experienced hand who would accept the responsibility of a forecast, and Mr. Thies has done so in the presence of our representative, not because he forgets that the Bill will be much altered by discussion in the House and moderated in Committee, but because he feels certain that the sooner its proposals are actively discussed by hospital managers and medical officers the better.

Asked as to his opinion on the Bill in general, Mr. Thies said:—

"The great scheme of State Insurance against sickness, introduced into the House of Commons by Mr. Lloyd George, which has been generally approved, quite irrespective of the usual party lines, is undoubtedly one of the boldest measures of social reform that has ever been introduced into Parliament by a responsible Minister of the Crown. Do not forget," Mr. Thies continued, "the Bill will affect some fourteen million workers, men, women, and children, insuring them against sickness; the workers, their employers, and the Government will each contribute towards the cost of carrying out the scheme."

"How do you think the Bill will be carried out?"

"It is highly probable that this proposed legislation will be carried out on the main lines indicated by the Chancellor of the Exchequer. Although it is too early to forecast all its effects, there can be no doubt that it must ultimately have a great influence upon the voluntary hospitals."

### The Bill and the Capitalist Subscriber.

"What will be its immediate effects?"

"It is too early to express any very definite opinion, but I may venture to say that, in the first place it is quite certain that employers of labour, who under this scheme will be compelled to contribute towards the insurance of all their employees, will not feel under any obligation to subscribe, as many of them do at the present time, towards the support of the voluntary hospitals. It seems certain that the great railway companies and the larger employers of labour will feel compelled to withdraw their present contributions, and this will have a serious effect upon the finances of these institutions."

### Its Effect on Out-Patients.

"Apart from finances, will the work of the voluntary hospitals be greatly affected?"

"It is difficult to estimate the effect of the scheme upon the work of the voluntary hospitals, inasmuch as provision is to be made for free medical attendance and medicines for the working classes, who, at the present time, make up the larger proportion of those attending the out-patient departments, so it may be anticipated that the numbers of such applicants for out-patient treatment will in the near future be very considerably diminished."

"And as regards the in-patients?"

"There again the question arises as to the position of voluntary hospitals in respect of the ward-patients, who at present are received without any charge being made towards the cost of their maintenance. Even at present many of these patients, who are being treated in the hospitals when suffering from the results of injuries received in their employment, are frequently receiving from their employers and their sick clubs a much larger weekly amount than they had previously received in wages when in full work; these patients receive free treatment at the hospitals, and, generally speaking, make no contribution whatever towards their maintenance."

### Malingering Cases.

"You mean this leads to malingering?"

"I mean that already, owing to the effects of the Employers' Liability Act, there is a tendency to malinger, and where, as is the case with some railways, the employees are compelled to join a sick-benefit society, the allowance from which, together with payments made by the employers, means more in sickness than in work, no injured man is likely under such conditions to get well faster than he can."

"You think malingering will increase?"

"The temptation may not be greater, but the numbers subject to it will be. At any rate, under the proposed scheme it is evident that the absence of any contribution to the hospitals from those who in sickness are well provided for cannot continue, and that some arrangement will have to be made whereby the hospitals shall be remunerated to some extent at least for the services which they thus render to the workers in cases of sickness."

"How do you think this would be done?"

### The Inevitable Result.

"One is led inevitably to the conclusion that in some way or other the voluntary hospitals must be subsidised either by the State or Municipalities."

"Do you think that would be a bad thing?"

"Bad or not, it exists already, both directly or indirectly. I can give you several examples. The London County Council is now giving grants-in-aid to those hospitals which have agreed to assist medically a specified number of school children. The Board of Education also subsidises a certain number of medical schools attached to hospitals, rightly holding that they are providing technical education quite as much as the previously aided polytechnics. The Poor-law, again, sends to the voluntary hospitals many of its more serious cases, and pays for the treatment accorded to them. As for indirect examples, the local authorities frequently reduce their assessments on hospitals for rating purposes, on the ground that the hospitals are doing an important work for the sick poor.''

A Concluding Suggestion.

'' Another aspect of the question which deserves careful consideration is the effect of the allowance of 30s. to all maternity cases upon the maternity departments of the hospitals. I cannot dogmatise at present as to the effects of that. But it leads me to throw out a suggestion I should like very much to see acted upon. The whole of the proposals are of such vital importance to the voluntary hospitals that a conference of hospital managers ought to be called with as little delay as possible for the purpose of freely considering the Chancellor of the Exchequer's proposals, and with a view to making suggestions bearing upon the effect of the scheme upon the voluntary hospitals. That is the principal constructive moral I can draw for you at present.''

